# Antihypertensive Deprescribing and Cardiovascular Events Among Long-Term Care Residents

**DOI:** 10.1001/jamanetworkopen.2024.46851

**Published:** 2024-11-25

**Authors:** Michelle C. Odden, Laura A. Graham, Xiaojuan Liu, Chintan V. Dave, Sei J. Lee, Yongmei Li, Bocheng Jing, Kathy Fung, Carmen A. Peralta, Michael A. Steinman

**Affiliations:** 1 Geriatric Research Education and Clinical Center, VA Palo Alto Health Care System, Palo Alto, California; 2 Department of Epidemiology and Population Health, Stanford University, Stanford, California; 3 Health Economics Resource Center, VA Palo Alto Health Care System, Palo Alto, California; 4 Center for Pharmacoepidemiology and Treatment Science, Institute for Health, Health Care Policy and Aging Research, Rutgers University, New Brunswick, New Jersey; 5 Department of Pharmacy Practice & Administration, Ernest Mario School of Pharmacy, Rutgers University, Piscataway, New Jersey; 6 Department of Veterans Affairs–New Jersey Health Care System, East Orange; 7 Division of Geriatrics, Department of Medicine, University of California San Francisco; 8 Geriatrics, Palliative, and Extended Care Service Line, San Francisco VA Medical Center, San Francisco, California; 9 Kidney Health Research Collaborative, University of California San Francisco and San Francisco VA Medical Center, San Francisco; 10 Habitat Health, San Francisco, California

## Abstract

**Importance:**

The practice of deprescribing antihypertensive medications is common among long-term care residents, yet the effect on cardiovascular outcomes is unclear.

**Objective:**

To compare the incidence of hospitalization for myocardial infarction (MI) or stroke among long-term care residents who are deprescribed or continue antihypertensive therapy.

**Design, Setting, and Participants:**

This comparative effectiveness research study used target trial emulation with observational electronic health record data from long-term care residents aged 65 years or older admitted to US Department of Veterans Affairs community living centers between October 1, 2006, and September 30, 2019, and taking at least 1 antihypertensive medication. Analyses were conducted between August 2023 and August 2024.

**Exposure:**

A reduction in the number of antihypertensive medications or dose (by ≥30%), assessed using barcode medication administration data.

**Main Outcome and Measures:**

Incidence of MI and stroke hospitalization up to 2 years was assessed using *International Classification of Diseases, Ninth Revision* and *International Statistical Classification of Diseases and Related Health Problems, Tenth Revision* codes. A pooled logistic regression model with inverse probability of treatment weighting (IPTW) and inverse probability of censoring weighting (IPCW) was used to estimate per-protocol effects.

**Results:**

Of 13 096 long-term care residents (97.4% men; median age, 77 years [IQR, 70-84 years]) taking antihypertensive medication, 17.8% were deprescribed antihypertensive medication over a period of 12 weeks. The estimated unadjusted cumulative incidence of stroke or MI hospitalization over 2 years was similar among residents who were and were not deprescribed antihypertensives in per-protocol analyses (11.2% vs 8.8%; difference, 2.4 percentage points [95% CI, −2.3 to 7.1 percentage points]). Participant characteristics were balanced after applying IPTW and IPCW; all standardized mean differences were less than 0.05. After full adjustment for confounding and informative censoring, the per-protocol analysis results showed no association of antihypertensive deprescribing with MI or stroke hospitalization (hazard ratio, 0.93; 95% CI, 0.70-1.26).

**Conclusions and Relevance:**

In this comparative effectiveness research study, deprescribing antihypertensive medication was not associated with risk of hospitalization for MI or stroke in long-term care residents. These findings may be informative for long-term care residents and clinicians who are considering deprescribing antihypertensive medications.

## Key Points

**Question:**

Is antihypertensive medication deprescribing associated with myocardial infarction and stroke in long-term care residents?

**Findings:**

In this comparative effectiveness research study using target trial emulation and including 13 096 US veterans residing in long-term care, no association between antihypertensive medication deprescribing and hospitalization for myocardial infarction or stroke was found.

**Meaning:**

These findings may be informative for long-term care residents and clinicians who are considering deprescribing antihypertensive medications.

## Teaser

This comparative effectiveness research study uses target trial emulation to assess whether deprescribing of antihypertensive medications is associated with hospitalization for myocardial infarction and stroke in long-term care residents.

## Introduction

Hypertension management in long-term care residents remains a challenging clinical problem. On one hand, the SPRINT trial demonstrated a benefit of intensive blood pressure (BP) control on a composite cardiovascular outcome even among older adults and those with frailty.^1^ On the other hand, long-term care residents have been excluded from most trials of BP lowering, and the presence of multiple chronic conditions as well as diminished physical and cognitive function could alter the risks of hypertension treatment.^2,3^ Moreover, among persons with limited life expectancy, the relative benefits of hypertension treatment may be outweighed by the harms.^4^ This has motivated clinicians’ and their patients’ interest in deprescribing, which is the planned and supervised act of discontinuing or reducing a medication when it is no longer beneficial. The decision of whether to deprescribe antihypertensive medication is complex and depends on patient preferences as well as the best available evidence on the potential benefits and harms.^5^

Our group demonstrated that deprescribing and medication changes were common among long-term care residents, with over 70% of US Department of Veterans Affairs (VA) long-term care residents receiving antihypertensive treatment having 1 or more medications deprescribed during their stay.^6^ Yet, the consequences of deprescribing remain poorly understood, especially for clinical cardiovascular events, which require a large sample size and long follow-up time to observe. As highlighted by a Cochrane review,^7^ the clinical utility of the current body of literature on antihypertensive deprescribing is constrained by limitations of existing studies, including short study duration and small sample size. Although the point estimate indicated greater risk of myocardial infarction (MI) (odds ratio [OR], 1.86; 95% CI, 0.19-17.98) and stroke (OR, 1.44; 95% CI, 0.25-8.35) following deprescribing of hypertensive medications, the estimate was imprecise and evidence quality was judged as low to very low due to the small number of trials, limited follow-up duration of 56 weeks or shorter, and the potential for bias. Thus, the consequences of deprescribing in terms of risk of MI or stroke remain indeterminate, especially within nursing home populations, where patient complexity and vulnerability often shift the risk-benefit calculus.

In the present study, we used target trial emulation methods to estimate the association of antihypertensive deprescribing with the risk of hospitalization for MI or stroke in VA long-term care residents. Target trial emulation integrates a set of design and analytic principles that attempt to emulate a randomized clinical trial based on observational data. This approach is recommended when clinical trial data are not available or feasible to collect.^8^ We used data from the VA because it represents a setting where nursing home data are linked with inpatient and outpatient care, allowing us to build a retrospective cohort study of long-term care residents and their engagement with the health care system.

## Methods

### Study Design and Population

In this comparative effectiveness research study, we used the International Society for Pharmacoeconomics and Outcomes Research (ISPOR) task force reporting guideline to inform our study design and analysis.^9^ This study received institutional review board (IRB) approval from Stanford University and the VA Palo Alto Health Care System, with a waiver of informed consent because the investigation involved minimal risk, the privacy notice informed patients that their records may be used without their authorization if approved by the IRB, study procedures were in place to protect confidentiality, information gleaned during the study did not affect the participants’ welfare, and the research could not be carried out without the waiver.

To emulate a hypothetical trial of deprescribing antihypertensive medication compared with continued use, we created an electronic cohort of residents admitted to a community living center (CLC), the VA equivalent of a nursing home. Residents were included if they were admitted between October 1, 2006, and September 30, 2019. Residents were excluded if they (1) were younger than 65 years at admission, (2) had a CLC stay less than 90 days (died or discharged during this time) to exclude those undergoing postacute rehabilitation, (3) had an acute hospital stay lasting more than 30 days any time during their CLC stay, (4) were not taking antihypertensive medications at admission, (5) had a history of heart failure or metastatic cancer at admission, (6) had systolic blood pressure greater than 160 mm Hg at the time of deprescribing, (7) were missing gender data as ascertained from the electronic health record, or (8) were residents in low-volume nursing homes (eFigure 1 in Supplement 1). Community living centers provide both long-term and short-term care, including rehabilitation; the restriction to stays of 90 or more days was intended to identify residents who received long-term care. The target trial baseline began at week 13 (91 days) to eliminate the possibility of immortal time bias due to this criterion. Immortal time bias occurs when there is a period of the study time in which death cannot occur; by requiring residents to stay at least 90 days to be included in this cohort, we ensured that residents survived this period. Thus, it is important that our study began after this period. In intent-to-treat (ITT) analysis, follow-up was censored at discharge, death, entry into hospice, or 2 years after admission. In per-protocol analysis, follow-up was additionally censored when a resident left their assigned treatment group—for example, when a person in the deprescribing group had a medication restarted or increased or when a person in the stable user group was deprescribed a medication. The per-protocol estimates were our primary estimates of interest because this approach estimates the effect of deprescribing if residents remained in their treatment groups. The ITT analysis estimated the effect of deprescribing, regardless of whether or not a resident stayed deprescribed. The protocol is outlined in Table 1.

**Table 1.** Target Trial Protocol

Protocol component	Target trial	Emulation
Eligibility criteria	VA nursing home resident aged ≥65 y at CLC admission Admitted to CLC between October 1, 2006, and September 30, 2019 Had a CLC stay ≥90 days (to identify residents who were admitted for long-term care) Had no acute hospital stays >30 days during CLC stay Taking antihypertensive medications at admission Systolic BP <160 mm Hg No history of heart failure	Criteria 1-7 (except criterion 3) were the same as the target trial; for criterion 3, the target trial began at week 13 to eliminate the possibility of immortal time bias; residents at a low-volume CLC and those missing data on gender were excluded
Treatment strategies	Initiate antihypertensive deprescribing and maintain for 2 wk (deprescribed group) vs stable and/or increased antihypertensive use (nondeprescribed group)	Same as the target trial
Treatment assignment	Individuals were randomly assigned to a strategy at baseline	Individuals were assigned according to the strategy with which their data were comparable at baseline, and an attempt was made to emulate randomization by accounting for confounding and deviation from the assigned group
Outcome	Time to adjudicated MI or stroke	Time to MI or stroke hospitalization, based on EHR
Follow-up period	Follow up residents for 2 y from baseline or until discharge, death, loss to follow-up, or March 1, 2022	Same as the target trial
Causal contrasts of interest	Intent-to-treat effect and per-protocol effect	Observational analogue of intent-to-treat and per-protocol effect
Analysis plan	Pooled logistic regression analysis of time to event compared across treatment strategies	Same as for the target trial, with additional adjustment for baseline covariates and preinitiation covariates using inverse probability weighting for treatment and adjustment for precensoring covariates using inverse probability weighting for censoring

Abbreviations: BP, blood pressure; CLC, community living center; EHR, electronic health record; MI, myocardial infarction; VA, US Department of Veterans Affairs.

For the purposes of target trial emulation, each week between weeks 13 and 24 was treated as an individual trial, collectively comprising a series of up to 12 trials. This approach is commonly used to increase precision of the estimates since the number of residents who change treatment in a given week is likely to be small.^8^ Residents were assessed for eligibility and split into 2 groups each week (time 0 for each trial): deprescribed vs not deprescribed. Residents were allowed to contribute multiple times.

The VA supports a national electronic health record that links all patients receiving care at all VA facilities. The primary data source for this study was the VA Corporate Data Warehouse (CDW), which is a national repository of data from the electronic health record, and CLC stays were identified using validated methods.^10,11^ This repository includes information on all inpatient and outpatient episodes of care within the VA and in the community setting, including related diagnoses and procedures, vital signs, laboratory values, and medications.^12,13,14^

### Treatment

Data on antihypertensive medication use were captured from the barcode medication administration system, which records medication administrations at a VA facility, including in CLC and acute care hospital stays. Antihypertensive medications were identified by the VA Drug Classification code. We included orally administered β-blockers, calcium channel blockers, angiotensin-converting enzyme inhibitors, angiotensin receptor blockers, central α-blockers, vasodilators, and loop, thiazide, and potassium-sparing diuretics.

We averaged antihypertensive medication use over 1-week periods to account for the fact that some medications were occasionally refused or held. Mean medication use was rounded to the nearest integer such that a medication given less than half of the days in a week would be considered not given. A deprescribing event was defined as a reduction in the number of antihypertensive medications or a reduction in dose of 30% or greater compared with the previous week and sustained for at least 2 weeks.^6^

### Outcomes

Outcomes of interest included hospitalization with MI or ischemic or hemorrhagic stroke (referred to collectively as cardiovascular events) that included an emergency department visit. Coding algorithms were based on prior literature and author opinion.^15^ Myocardial infarction events were identified based on hospital discharge records using *International Classification of Diseases, Ninth Revision (ICD-9)* code 410.x or *International Statistical Classification of Diseases and Related Health Problems, Tenth Revision (ICD-10)* codes I21.x or 122.x in any position. A prior study in the VA found that the positive predictive value (PPV) of acute MI in the first position of hospital discharge records was 96.9%.^16^ Stroke events were also based on hospital discharge records using *ICD-9* codes 430, 431, 433, 434, or 436 and *ICD-10* codes I60, I61, I63, or I64 in any position. A recent VA study found that most of these stroke codes had a PPV of 80% or higher for stroke hospitalization; the most commonly used code (*ICD-10* I63) had a PPV of 90%.^17^ As a secondary outcome, we used a broader definition of coronary heart disease and cerebrovascular events by including unstable angina (eTable 1 in Supplement 1) and transient ischemic attack (eTable 1 in Supplement 1) in addition to MI and stroke events. Urinary tract infection (UTI) was used as a negative control outcome (eTable 1 in Supplement 1).

### Covariates

The CDW Patient domain, which extracts data from the patient’s electronic health record, was used to determine age, gender, race, and ethnicity at the time of the CLC admission. Race and ethnicity were included in the analysis to account for the fact that prescribing patterns and the risk of hospitalization for MI and stroke may differ across racial and ethnic groups. Racial categories included American Indian or Alaska Native, Asian or Pacific Islander, Black, White, and multiracial. Ethnicity categories included Hispanic or not Hispanic. Locality was categorized as 1 of 5 geographic regions of the US: Pacific, Continental, Midwest, Southeast, and North Atlantic.^18^ Diagnosis codes and laboratory values were obtained from the CDW Inpatient and Outpatient domains. Chronic conditions, including indications for antihypertensive use and other cardiovascular conditions (including prior stroke and MI), were identified using *International Classification of Diseases, Ninth Revision, Clinical Modification* and *International Statistical Classification of Diseases, Tenth Revision, Clinical Modification* diagnosis codes 1 year prior to and during the nursing home stay.^15^ Body mass index was calculated based on recorded height and weight in the CDW. Data on cognitive function, activities of daily living, and falls were obtained from the Centers for Medicare & Medicaid Services Minimum Data Set, which changed from version 2.0 to 3.0 on October 1, 2010. We used the Cognitive Function Scale to combine the cognitive assessment tools from Minimum Data Set 2.0 and 3.0 into a single, integrated, 4-level assessment of cognitive function: cognitively intact, mildly impaired, moderately impaired, and severely impaired.^19^ Systolic and diastolic BP measurements were ascertained from the CDW Vital Signs domain. All variables were available as time varying with the exception of race, ethnicity, and locality. Missing data were limited (eTable 2 in Supplement 1); nonetheless, we used a missing indicator in analyses so that participants with missing data would not be dropped from the analytic sample.

### Statistical Analysis

We first described the proportion of residents who met our definition of deprescribing antihypertensive treatment in weeks 13 to 24 and characterized their measurements either at admission or, for time-varying measures, at the most recent measure. We plotted Kaplan-Meier survival curves and used a log-rank test to assess statistically significant differences by treatment group. We used unadjusted pooled logistic regression to estimate the hazard ratio (HR) of cardiovascular events among residents who were deprescribed antihypertensives compared with those who were not.

We next ran a pooled logistic regression model of deprescribing (treatment model) at weeks 13 to 24 as a function of baseline and time-varying pretreatment covariates listed in Table 2 and modeled time with a cubic spline term. We evaluated the positivity assumption by plotting the probability of treatment by observed treatment status. We calculated the stabilized inverse probability of treatment weights, and the final pooled logistic regression model (outcome model) was weighted by these weights to estimate the association of deprescribing with cardiovascular events in the ITT analysis. We also adjusted for baseline and time-varying covariates within the pooled logistic regression (outcome model) to reduce residual confounding.

**Table 2.** Characteristics of Long-Term Care Residents Overall and by Deprescribing Group

Characteristic	Residents^a^		
	Overall (N = 13 096)	Not deprescribed (n = 10 762 [82.2%])	Deprescribed (n = 2334 [17.8%])
Age at admission, median (IQR), y	77 (70-84)	77 (70-84)	77 (70-84)
Gender			
Men	12 759 (97.4)	10 483 (97.4)	2276 (97.5)
Women	337 (2.6)	279 (2.6)	58 (2.5)
Race			
American Indian or Alaska Native	62 (0.5)	52 (0.5)	10 (0.4)
Asian or Pacific Islander	150 (1.1)	118 (1.1)	32 (1.4)
Black	2424 (18.5)	1987 (18.5)	437 (18.7)
White	9449 (72.2)	7774 (72.2)	1675 (71.8)
Multiracial	102 (0.8)	76 (0.7)	26 (1.1)
Missing data	909 (6.9)	755 (7.0)	154 (6.6)
Ethnicity			
Hispanic	591 (4.5)	487 (4.5)	104 (4.5)
Non-Hispanic	12 505 (95.5)	10 275 (95.5)	2230 (95.5)
Baseline height			
Median (IQR), m	1.8 (1.7-1.8)	1.8 (1.7-1.8)	1.8 (1.7-1.8)
Unknown, No.	855	735	120
Baseline weight			
Median (IQR), kg	82.1 (71.2-95.7)	82.1 (71.2-95.7)	82.6 (72.1-96.2)
Unknown, No.	7	5	2
Blood pressure, median (IQR), mm Hg			
Systolic	129 (119-139)	129 (119-139)	127 (117-138)
Diastolic	70 (65-76)	70 (65-76)	69 (64-74)
Standardized daily dose in first week, median (IQR)^b^	1.00 (0.50-2.50)	1.00 (0.50-2.45)	1.29 (0.50-3.00)
Daily hypertension medications in first week, median, No.			
1	6685 (51.0)	5772 (53.6)	913 (39.1)
2	4057 (31.0)	3228 (30.0)	829 (35.5)
3	1658 (12.7)	1262 (11.7)	396 (17.0)
>3	696 (5.3)	500 (4.6)	196 (8.4)
Antihypertensive medication use at time 0			
ACE inhibitors or ARBs	5734 (43.8)	4589 (42.6)	1145 (49.1)
β-Blockers	6898 (52.7)	5450 (50.6)	1448 (62.0)
Calcium channel blockers	4537 (34.6)	3702 (34.4)	835 (35.8)
Diuretics	3484 (26.6)	2761 (25.7)	723 (31.0)
Other	1186 (9.1)	903 (8.4)	283 (12.1)
CFS score at baseline^c^			
1	3131 (23.9)	2581 (24.0)	550 (23.6)
2	5116 (39.1)	4183 (38.9)	933 (40.0)
3	3206 (24.5)	2637 (24.5)	569 (24.4)
4	1077 (8.2)	895 (8.3)	182 (7.8)
Missing	566 (4.3)	466 (4.3)	100 (4.3)
ADLs at baseline			
Median (IQR), No.	14 (8-20)	14 (8-20)	14 (8-20)
Unknown, No.	45	39	6
Experienced falls in the first 30 d	68 (0.5)	55 (0.5)	13 (0.6)
Comorbidities			
Diabetes	6522 (49.8)	5289 (49.1)	1233 (52.8)
Osteoarthritis	4416 (33.7)	3606 (33.5)	810 (34.7)
Chronic obstructive pulmonary disease	2061 (15.7)	1687 (15.7)	374 (16.0)
Atrial fibrillation	2903 (22.2)	2326 (21.6)	577 (24.7)
Coronary heart disease	5218 (39.8)	4203 (39.1)	1015 (43.5)
Cerebrovascular disease	5669 (43.3)	4590 (42.7)	1079 (46.2)
Peripheral vascular disease	4120 (31.5)	3296 (30.6)	824 (35.3)
Depression	6890 (52.6)	5620 (52.2)	1270 (54.4)
Any malignant neoplasm at admission^d^	3264 (24.9)	2634 (24.5)	630 (27.0)
Kidney failure	3851 (29.4)	3025 (28.1)	826 (35.4)
Acute kidney injury	3479 (26.6)	2743 (25.5)	736 (31.5)
Trials, median (IQR), No.	9 (4-12)	11 (4-12)	4 (2-7)
Follow-up time, median (IQR), wk			
ITT	20 (6-72)	19 (6-73)	22 (10-66)
PP	12 (5-29)	13 (5-32)	8 (4-16)

Abbreviations: ACE, angiotensin-converting enzyme; ADL, activity of daily living; ARB, angiotensin receptor blocker; CFS, Cognitive Function Scale; ITT, intent to treat; PP, per protocol.

^a^ Data are presented as number (percentage) of participants unless otherwise indicated.

^b^ Specified as the usual maintenance dose in reference pharmacopoeias.

^c^ Scores range from 1 to 4, with higher scores indicating worse cognitive impairment.

^d^ Includes lymphoma and leukemia.

For per-protocol analyses, we next evaluated medication changes after time 0 and censored residents who left their treatment group. We again used pooled logistic regression to model the probability of being censored (censoring model) as a function of precensoring covariates and time. The inverse probabilities of censoring weights were calculated, and we multiplied these by the inverse probability of treatment weights to estimate the association of deprescribing with cardiovascular events. As a sensitivity analysis, we applied the inverse probability of censoring due to mortality weights to account for differential mortality between the 2 arms, and we also censored follow-up time at 6 months. We additionally ran analyses with a broader definition of coronary heart disease and cerebrovascular disease outcomes. Finally, we assessed for the presence of effect modification by dementia status, BP thresholds of 140 mm Hg (systolic) and 80 mm Hg (diastolic), and linear age.

Analyses were conducted between August 2023 and August 2024. Statistical significance was based on 2-sided *P* ≤ .05. All analyses were conducted in R, version 4.3.2 (R Project for Statistical Computing).

## Results

We identified 13 096 residents who met our inclusion or exclusion criteria (eFigure 1 in Supplement 1); 97.4% were men, 2.6% were women, and the median age was 77 years (IQR, 70-84 years). A total of 4.5% were Hispanic, and 95.5% were non-Hispanic ethnicity; 0.5% were American Indian or Alaska Native, 1.1% were Asian or Pacific Islander, 18.5% were Black, 72.2% were White, 0.8% were multiracial, and 6.9% had missing race data. Overall, each resident contributed to a median of 10 trials (IQR, 4-12 trials). The population had a high burden of chronic conditions; the most common conditions were diabetes (49.8%), coronary heart disease (39.8%), cerebrovascular disease (43.3%), and depression (52.6%) (Table 1). In the course of 12 weeks, 2334 residents (17.8%) were deprescribed antihypertensive medication. Those who were deprescribed had slightly lower systolic BP and diastolic BP and were taking more antihypertensive medications (Table 2). Residents with a history of diabetes, coronary heart disease, cerebrovascular disease, peripheral vascular disease, kidney failure, and acute kidney injury were more likely to be deprescribed antihypertensives.

The treatment model demonstrated good overlap in the probability of treatment and probability of censoring in the deprescribed and not deprescribed groups (eFigure 2 in Supplement 1). Resident characteristics were balanced after inverse probability of treatment and inverse probability of censoring weighting; all standardized mean differences were less than 0.05 (eFigure 3 in Supplement 1). The inverse probability of censoring weights also resulted in an attenuation of differences among residents who were and who were not censored due to death (eFigure 4 in Supplement 1).

In unadjusted analyses, the estimated cumulative incidence of stroke or MI hospitalizations over 2 years (median follow-up: 20 weeks) was higher in the deprescribing group in the ITT analysis (11.7% vs 9.4%; difference, 2.3 percentage points [95% CI, −0.2 to 4.8 percentage points]). This difference was attenuated in the per-protocol analysis (11.2% vs 8.8%; difference, 2.4 percentage points [95% CI, −2.3 to 7.1 percentage points]) (Figure). There were 6879 residents (52.5%) censored in the per-protocol analysis at the time that they deviated from their treatment group assigned at time 0; median time to censoring was 12 weeks (IQR, 5-29 weeks). In ITT analysis, follow-up was censored at discharge (7041 of 13 096 residents [53.8%]), death (3021 [23.1%]), entry into hospice (52 [0.4%]), or 2 years after admission (2410 [18.4%]).

**Figure.**
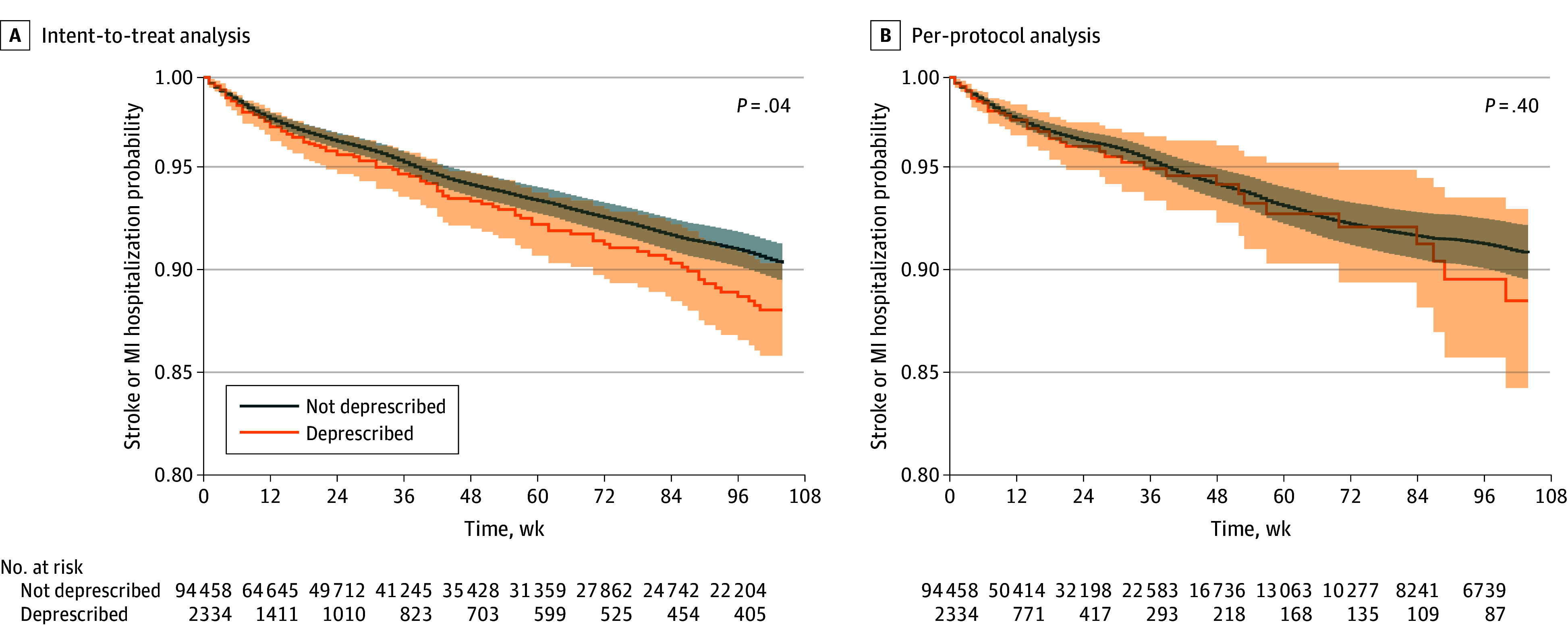
Unadjusted Kaplan-Meier Plots of Hospitalization for Myocardial Infarction (MI) or Stroke in Intent-to-Treat and Per-Protocol Analyses Shading indicates 95% CIs.

The unadjusted HR for stroke or MI hospitalization following deprescribing was 1.21 (95% CI, 1.01-1.47) in the ITT analysis and 1.15 (95% CI, 0.88-1.51) when censored as per protocol. After adjusting for confounding using the inverse probability of treatment weights, the ITT HR estimates attenuated to 1.03 (95% CI, 0.83-1.27). For the primary analysis (ie, per protocol), after fully adjusting for confounding and informative censoring, the HR was attenuated to 0.93 (95% CI, 0.71-1.23) (Table 3). As a sensitivity analysis, we additionally weighted by the inverse probability of censoring due to death, and the HR was similar (0.86; 95% CI, 0.63-1.18). Results were similar when we censored the follow-up at 6 months (HR, 0.97; 95% CI, 0.68-1.39).

**Table 3.** HRs of Deprescribing Antihypertensive Medication and MI or Stroke Hospitalization Based on Target Trial Emulation

Outcome	Intent-to-treat analysis			Per-protocol analysis		
	Event rate, per 1000 person-years		HR (95% CI)	Event rate, per 1000 person-years		HR (95% CI)
	Deprescribed	Not deprescribed		Deprescribed	Not deprescribed	
MI or stroke						
Overall	69.6	55.9	NA	75.9	62.1	NA
Unadjusted	NA	NA	1.21 (1.01-1.47)	NA	NA	1.15 (0.88-1.51)
IPTW^a^	NA	NA	1.03 (0.83-1.27)	NA	NA	0.99 (0.74-1.31)
IPTW plus IPCW^a^	NA	NA	NA	NA	NA	0.93 (0.71-1.23)
Expanded outcome definition^b^						
Overall	74.2	61.3	NA	81.6	67.4	NA
Unadjusted	NA	NA	1.18 (0.99-1.42)	NA	NA	1.14 (0.88-1.48)
IPTW^a^	NA	NA	1.01 (0.82-1.24)	NA	NA	0.97 (0.73-1.30)
IPTW plus IPCW^a^	NA	NA	NA	NA	NA	0.94 (0.70-1.26)
Urinary tract infection						
Overall	248.5	222.1	NA	283.2	237.7	NA
Unadjusted	NA	NA	1.09 (0.98-1.21)	NA	NA	1.12 (0.97-1.29)
IPTW^a^	NA	NA	1.01 (0.89-1.13)	NA	NA	1.01 (0.86-1.20)
IPTW plus IPCW^a^	NA	NA	NA	NA	NA	0.99 (0.84-1.17)

Abbreviations: HR, hazard ratio; IPCW, inverse probability of censoring weighting; IPTW, inverse probability of treatment weighting; MI, myocardial infarction; NA, not applicable.

^a^ Additionally adjusted for baseline systolic blood pressure, diastolic blood pressure, daily medication dose and number, weight, height, gender, ethnicity, race, Cognitive Function Scale score, activities of daily living, history of falls, age, comorbidities, and baseline laboratory values.

^b^ The expanded outcome definition includes MI, stroke, unstable angina, and transient ischemic attack.

Patterns were similar when we used a broader definition of coronary heart disease and cerebrovascular disease outcomes. The adjusted HR in per-protocol analysis was 0.94 (95% CI, 0.70-1.26) (Table 3).

There was no evidence of effect modification by age (*P* = .80 for interaction), dementia diagnosis (*P* = .90 for interaction), systolic BP greater than 140 mm Hg (*P* = .54 for interaction), or diastolic BP greater than 80 mm Hg (*P* = .67 for interaction) in per-protocol models. As a negative control outcome, the cumulative incidence of UTI at 1 year was 33% in the deprescribed group and 32% in the not deprescribed group. The adjusted HR using the ITT design for UTI following deprescribing was 1.01 (95% CI, 0.89-1.13) and for the primary per-protocol analysis was estimated as 0.99 (95% CI, 0.84-1.17) (Table 3).

## Discussion

In this comparative effectiveness research study, deprescribing antihypertensive medication did not appear to be associated with the risk of stroke or MI hospitalizations in a population of long-term care residents residing in VA nursing homes over 2 years of follow-up. Specifically, we did not find evidence to reject the null hypothesis of no difference in risk of stroke or MI hospitalization between those residents who were deprescribed antihypertensive treatment and those who continued antihypertensive treatment. However, we note that the precision of our estimates was limited, lending uncertainty to our findings. Our results are supported by analysis of a negative control outcome, UTI, for which there was no association with deprescribing after adjustment. In our study, over half of the residents (52.5%) did not maintain their antihypertensive medication regimen and were censored in per-protocol analysis. Nonetheless, neither ITT nor per-protocol estimates suggested an increase in cardiovascular event risk. These results may be informative for long-term care residents and clinicians who are considering deprescribing antihypertensive medications.

We presented our findings in comparison with a Cochrane review of antihypertensive medication withdrawal, which reported an OR for MI of 1.86 (95% CI, 0.19-17.98) and for stroke of 1.44 (95% CI, 0.25-8.35).^7^ However, the review noted the low quality of the evidence, including the short follow-up. Only 2 of the 5 studies included residents in a care setting; neither of the studies had any MI outcomes, and in 1, there was a single stroke in the discontinuation group.^20,21^ Moreover, these studies were only of diuretics and took place over 40 years ago.^20,21^ In addition to being a more contemporary study, the high degree of comorbidity and frailty in the population in our study could explain any differences, as well as the fact that the studies in the Cochrane review^7^ were trials and we conducted an emulated trial, which is subject to unmeasured confounding.

Previous observational research reported an increase in adverse outcomes among individuals taking multiple antihypertensive medications who had low BP. The PARTAGE study^22^ showed that 20% of persons with hypertension living in nursing homes had systolic BP levels less than 130 mm Hg while treated with more than 2 antihypertensive agents, and these persons had more than 2-fold higher risk of death compared with other persons with hypertension. Findings were similar for deaths due to cardiovascular events. Another study from our team found similar findings for cardiovascular events. Liu et al^23^ reported that among persons taking antihypertensive medications, a systolic BP less than 110 mm Hg was associated with a greater risk of MI and stroke, but this association was not present among persons taking no antihypertensive medications. Taken together, these findings support our observation that deprescribing antihypertensive medications was not associated with risk of MI or stroke.

The mechanisms mediating a null or beneficial effect of deprescribing antihypertensive medications among long-term care residents are uncertain. Although trial evidence has demonstrated a protective effect of BP lowering on cardiovascular outcomes,^1^ these benefits may be attenuated or even inverted in some long-term care residents. The long-term care population may be vulnerable to adverse effects from BP overtreatment because these patients tend to have a higher prevalence of functional limitation, polypharmacy, multimorbidity, and perhaps, differences in cerebral blood flow autoregulation compared with the general population.^24^ A recent trial, OPTIMAL-BP, of intensive vs conventional BP lowering after endovascular thrombectomy was stopped early due to an elevated risk of functional dependence in the intensive control group.^25^ That trial highlighted the risks of intensive BP lowering in a population with microvascular damage and the potential to exacerbate ischemic injury.

### Strengths and Limitations

Our study has many strengths, including the ability to link data from nursing home, inpatient, and outpatient visits as well as the use of barcode medication administration to characterize medication use. However, there are also limitations that should be considered when interpreting the findings. First, the 95% CIs of our results were wide, which does not exclude the possibility of risk for harm with either treatment group. We restricted our study population to those who stayed at the CLC for at least 90 days to identify a long-term care population, but this precluded us from examining deprescribing during this period. The biggest challenge of target trial emulation is the potential for unmeasured confounding. We included a wide range of clinical characteristics that could confound deprescribing; however, the possibility of unmeasured or residual confounding remains. In a clinical setting, treatment decisions are often made as a result of a number of complex and interwoven factors, and our results reflect the combined effects of deprescribing regardless of the reason. Additionally, we found that antihypertensive medication use was not stable over time; thus, a moderate proportion of residents did not remain in the assigned treatment group. By modeling both ITT and per-protocol effects, we presented estimates for the effects of deprescribing among those who stayed in or may have deviated from their treatment groups. Another limitation of this research is that the VA long-term care population is not representative of the general US long-term care population and includes a higher proportion of men. We do not know of any reason why the effects of deprescribing would differ among older men and women, but it should be investigated in future studies. Additionally, there is known misclassification in coding in the electronic health record, and this may have affected our results. We also did not look at differences in deprescribing different antihypertensive classes due to concerns about sample size and sufficient outcome events by group.

## Conclusions

In this comparative effectiveness research study, deprescribing antihypertensive medication was not associated with risk of stroke or MI hospitalization in long-term care nursing home residents over 2 years of follow-up. A randomized clinical trial would help address concerns about unmeasured confounding but could be challenging to implement in this complex population. We recognize that treatment decision-making requires a holistic evaluation of benefits and harms, and this study was limited to the study of cardiovascular disease hospitalizations. More research is needed on deprescribing methods and potential consequences to inform this growing practice and inform patient and clinician shared decision-making.

## Notes

Supplement 1. **eFigure 1.** Study Flow Diagram **eFigure 2.** Probability of Treatment Overlap, Censoring Overlap, Treatment Weights, and Censoring Weights by Deprescribed and not Deprescribed Groups **eFigure 3.** Standardized Mean Differences Before and After Applying Inverse Probability Weighting for Treatment and Censoring Due to Leaving Assigned Treatment Group **eFigure 4.** Standardized Mean Differences Before and After Applying Inverse Probability Weighting for Censoring Due to Mortality **eTable 1.** List of *ICD* Codes for Nonprimary Outcomes **eTable 2.** Missing Data by Resident-Weeks

Supplement 2.
Data Sharing Statement
