# Supplementary material for: Antihypertensive Deprescribing and Cardiovascular Events Among Long-Term Care Residents
Source: JAMA Netw Open. 2024 Nov 25;7(11):e2446851. doi: 10.1001/jamanetworkopen.2024.46851 (PMC11589794; doi:10.1001/jamanetworkopen.2024.46851)
Supplement: Supplement 1. — eFigure 1. Study Flow Diagram eFigure 2. Probability of Treatment Overlap, Censoring Overlap, Treatment Weights, and Censoring Weights by Deprescribed and not Deprescribed Groups eFigure 3. Standardized Mean Differences Before and After Applying Inverse Probability Weighting for Treatment and Censoring Due to Leaving Assigned Treatment Group eFigure 4. Standardized Mean Differences Before and After Applying Inverse Probability Weighting for Censoring Due to Mortality eTable 1. List of ICD Codes for Nonprimary Outcomes eTable 2. Missing Data by Resident-Weeks [file jamanetwopen-e2446851-s001.pdf]

Supplementary Online Content

Odden MC, Graham LA, Liu X, et al. Antihypertensive deprescribing and cardiovascular events among long-term care residents. *JAMA Netw Open*. 2024;7(11):e2446851. doi:10.1001/jamanetworkopen.2024.46851

eFigure 1. Study Flow Diagram

eFigure 2. Probability of Treatment Overlap, Censoring Overlap, Treatment Weights, and Censoring Weights by Deprescribed and not Deprescribed Groups

eFigure 3. Standardized Mean Differences Before and After Applying Inverse Probability Weighting for Treatment and Censoring Due to Leaving Assigned Treatment Group

eFigure 4. Standardized Mean Differences Before and After Applying Inverse Probability Weighting for Censoring due to Mortality

eTable 1. List of *ICD* Codes for Nonprimary Outcomes

eTable 2. Missing Data by Resident-Weeks

This supplementary material has been provided by the authors to give readers additional information about their work.

© 2024 Odden MC et al. *JAMA Network Open*.

eFigure 1: Study flow diagram

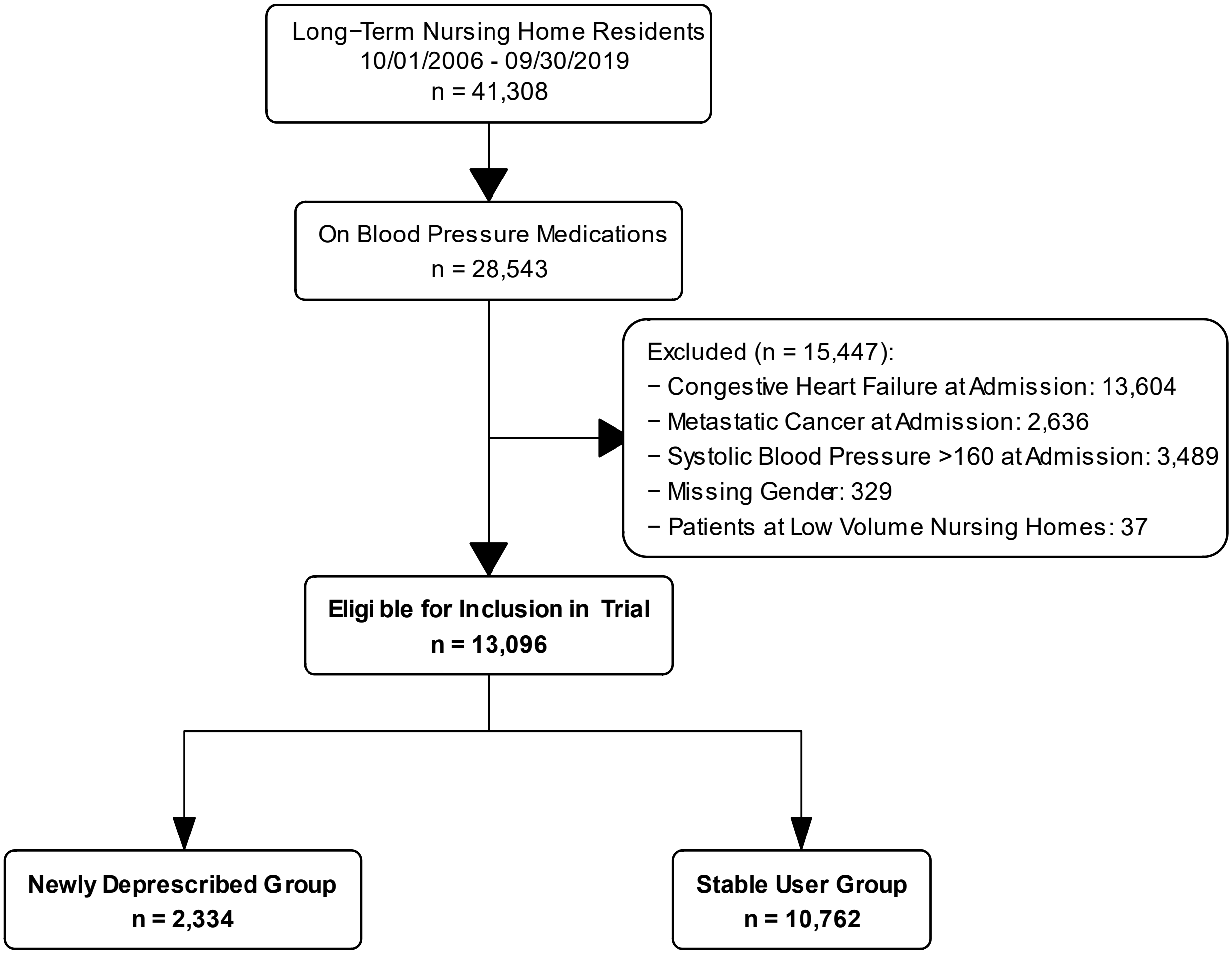

© 2024 Odden MC et al. *JAMA Network Open*.

eFigure 2: a) Probability of treatment overlap by deprescribed and not deprescribed groups, b) Probability of censoring (due to leaving assigned treatment group) overlap by deprescribed and not deprescribed groups, c) Treatment weights by deprescribing and not deprescribed groups, d) Censoring weights by deprescribing and not deprescribed groups

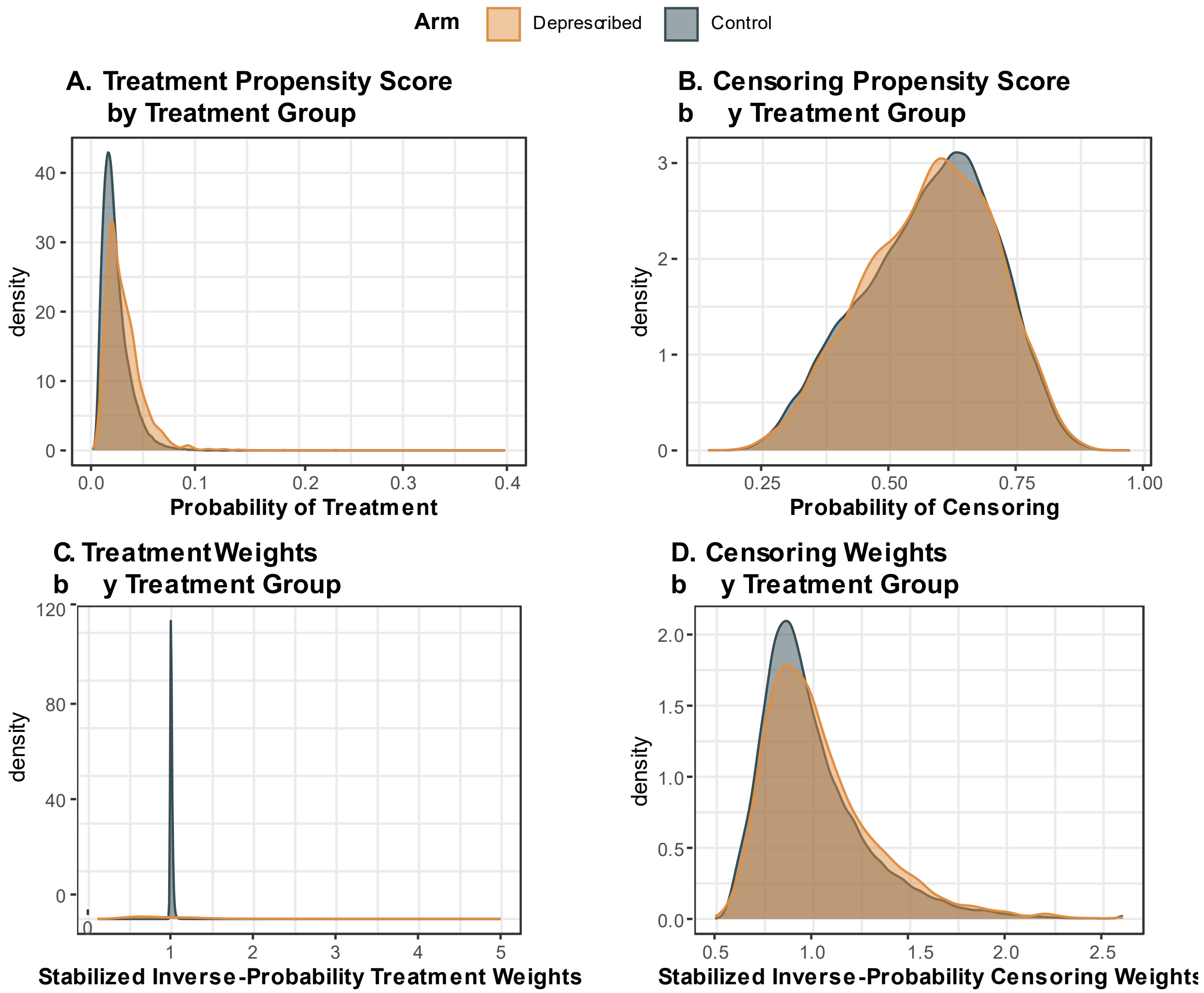

© 2024 Odden MC et al. *JAMA Network Open*.

eFigure 3: Standardized mean differences before and after applying inverse probability weighting for treatment (left) and censoring due to due to leaving assigned treatment group (right)

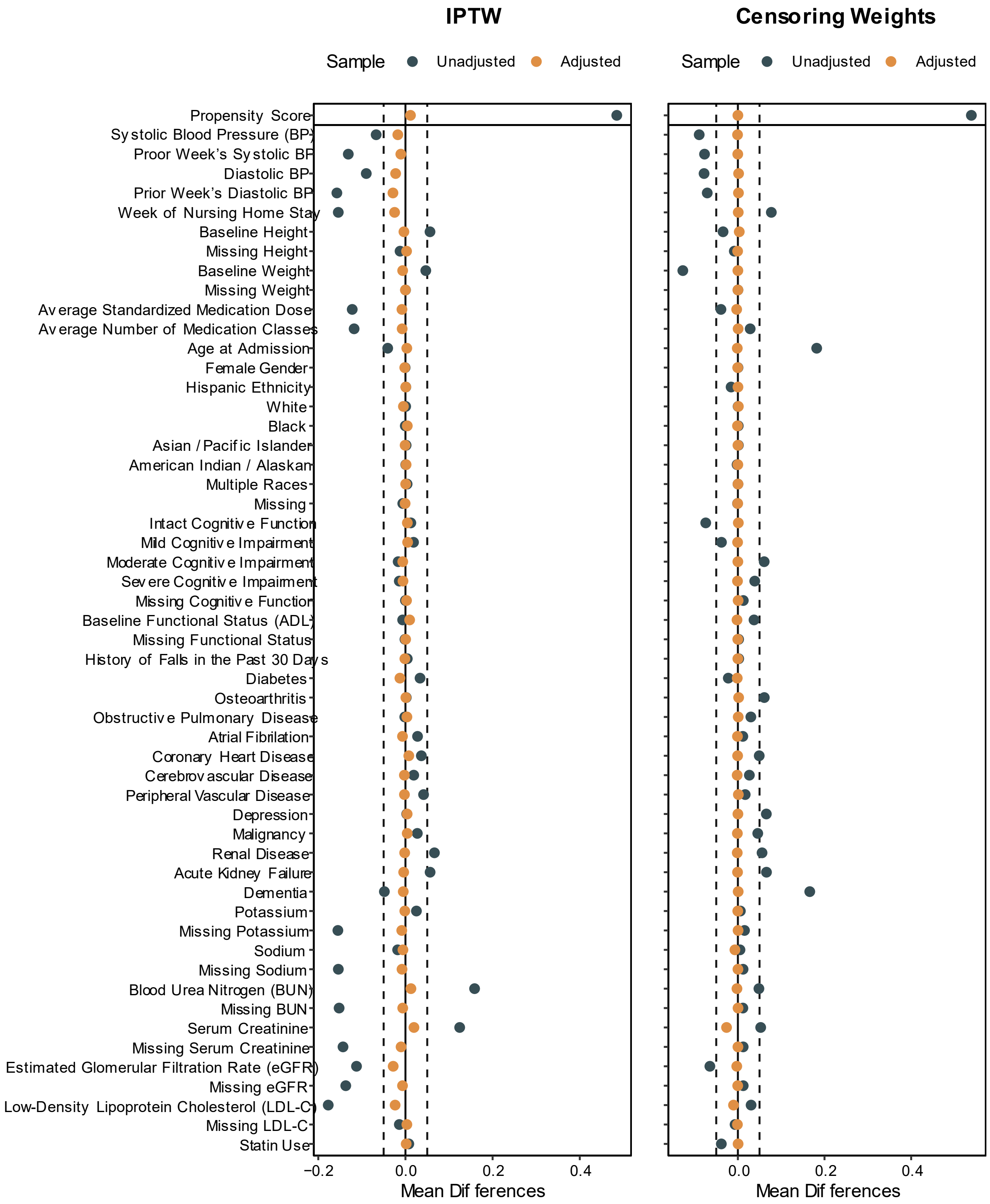

© 2024 Odden MC et al. *JAMA Network Open*.

eFigure 4: Standardized mean differences before and after applying inverse probability weighting for censoring due to mortality

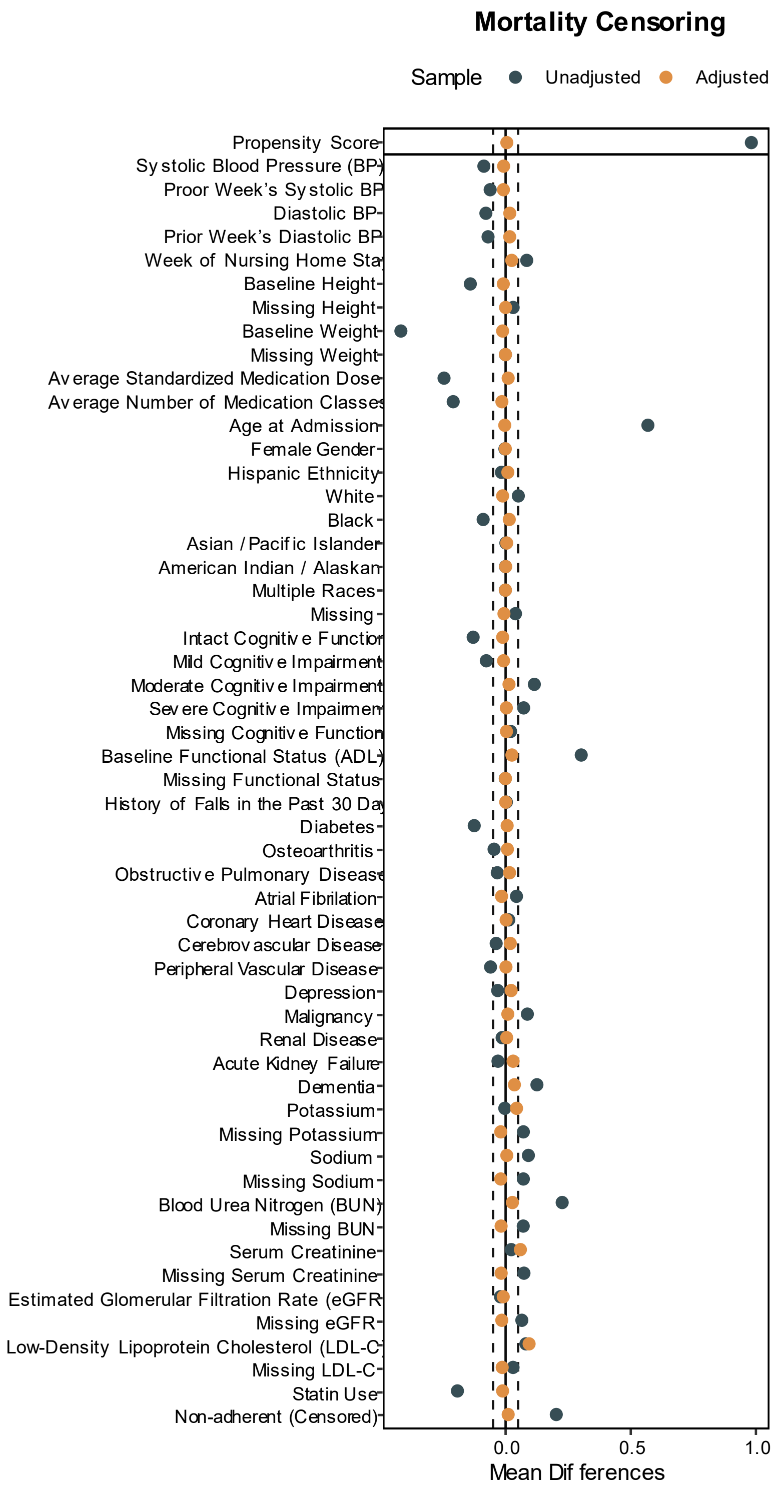

© 2024 Odden MC et al. *JAMA Network Open*.

eTable 1: List of ICD codes for non-primary outcomes

	ICD-9	ICD-10
Unstable Angina	411.1	I20.0, I25.700, I25.720, I25.110, I25.750, I25.710, I25.760, I25.790, I25.730
Transient Ischemic Attack	430, 431, 433.01, 433.11, 433.21, 433.31, 433.81, 433.91, 434.00, 434.01, 434.10, 434.11, 434.90, 434.91,<br><br>435.0, 435.1, 435.3, 435.8, 435.9, 436, 997.02	G45.0, G45.1, G45.2, G45.8, G45.9, G46.0, G46.1, G46.2, G46.3, G46.4, G46.5, G46.6, G46.7, G46.8, G97.31, G97.32, I60.00, I60.01, I60.02, I60.10, I60.11, I60.12, I60.20, I60.21, I60.22, I60.30, I60.31, I60.32, I60.4, I60.50, I60.51, I60.52, I60.6, I60.7, I60.8, I60.9, I61.0, I61.1, I61.2, I61.3, I61.4, I61.5, I61.6, I61.8, I61.9, I63.00, I63.011, I63.012, I63.013, I63.019, I63.02, I63.031, I63.032, I63.039, I63.09, I63.10, I63.111, I63.112, I63.113, I63.119, I63.12, I63.131, I63.132, I63.133, I63.139, I63.19, I63.20, I63.211, I63.212, I63.213, I63.219, I63.22, I63.231, I63.232, I63.233, I63.239, I63.29, I63.30, I63.311, I63.312, I63.313, I63.319, I63.321, I63.322, I63.323, I63.329, I63.331, I63.332, I63.333, I63.339, I63.341, I63.342, I63.343, I63.349, I63.39, I63.40, I63.411, I63.412, I63.413, I63.419, I63.421, I63.422, I63.423, I63.429, I63.431, I63.432, I63.433, I63.439, I63.441, I63.442, I63.443, I63.449, I63.49, I63.50, I63.511, I63.512, I63.513, I63.519, I63.521, I63.522, I63.523, I63.529, I63.531,

© 2024 Odden MC et al. *JAMA Network Open*.

		I63.532, I63.533, I63.539, I63.541, I63.542, I63.543, I63.549, I63.59, I63.6, I63.8, I63.81, I63.89, I63.9, I66.01, I66.02, I66.03, I66.09, I66.11, I66.12, I66.13, I66.19, I66.21, I66.22, I66.23, I66.29, I66.3, I66.8, I66.9, I67.841, I67.848, I67.89, I97.810, I97.811, I97.820, I97.821
Urinary Tract Infection	599.0, 996.64	N39.0, T83.510A, T83.511A, T83.512A, T83.518A

© 2024 Odden MC et al. *JAMA Network Open*.

eTable 2: Missing data by resident-weeks

**Characteristic**	**% of resident-weeks missing data**
Weight	0.2%
Height	5.7%
Race	6.9%
CFS	4.3%
ADL	0.4%
Potassium Lab	1.6%
Sodium Lab	1.0%
BUN Lab	1.4%
Creatinine Lab	4.5%
eGFR Lab	8.4%
LDLC Lab	23.0%

© 2024 Odden MC et al. *JAMA Network Open*.
