# Supplementary material for: Antihypertensive Deprescribing and Cardiovascular Events Among Long-Term Care Residents
Source: JAMA Netw Open. 2024 Nov 25;7(11):e2446851. doi: 10.1001/jamanetworkopen.2024.46851 (PMC11589794; doi:10.1001/jamanetworkopen.2024.46851)
Supplement: Supplement 2. — Data Sharing Statement [file jamanetwopen-e2446851-s002.pdf]

Data Sharing Statement

Odden. Antihypertensive Deprescribing and Cardiovascular Events Among Long-Term Care Residents. *JAMA Netw Open*. Published November 25, 2024.
doi:10.1001/jamanetworkopen.2024.46851

Data
Data available: No

Additional Information
Explanation for why data not available: VA policies prohibit data sharing except to VA employees with an approved research proposal.
